# Cytotoxicity of methanol extracts of 10 Cameroonian medicinal plants towards multi-factorial drug-resistant cancer cell lines

**DOI:** 10.1186/s12906-016-1253-3

**Published:** 2016-08-02

**Authors:** Victor Kuete, Cedric F. Tchinda, Flora T. Mambe, Veronique P. Beng, Thomas Efferth

**Affiliations:** 1 Department of Pharmaceutical Biology, Institute of Pharmacy and Biochemistry, University of Mainz, 55128 Mainz, Germany; 2 Department of Biochemistry, Faculty of Science, University of Dschang, P.O. Box 67, Dschang, Cameroon; 3 Department of Biochemistry, Faculty of Science, University of Yaounde I, Yaounde, Cameroon

**Keywords:** *Albizia adiathifolia*, *Alchornea cordifolia*, Apoptosis, Cameroon, Cancer, Cytotoxicity, Medicinal plants, Multidrug resistance

## Abstract

**Background:**

Cancer chemotherapy is still hampered by clinical failures due to multi-drug resistance (MDR) of tumor cells. In the present study, we have investigated the cytotoxicity of 20 methanol extracts from 10 medicinal plants against the sensitive leukemia CCRF-CEM cells. The most cytotoxic extracts were then further tested on a panel of 8 human cancer cell lines, including various MDR phenotypes.

**Methods:**

The cytotoxicity of the 20 methanol extracts from 10 Cameroonian medicinal plants was determined using a resazurin reduction assay. Meanwhile, flow cytometry was used to measure cell cycle, apoptosis, mitochondrial membrane potential (MMP), and reactive oxygen species (ROS).

**Results:**

In the preliminary assay using CCRF-CEM cells, 12 extracts from five plants displayed IC_50_ values below 80 μg/mL, namely *Albizia adianthifolia, Alchornea cordifolia, Alchornea laxiflora, Pennisetum purpureum,* and *Spathodea campanulata*. the four best extracts were from two plants: *Albizia adianthifolia* roots (AAR) and bark (AAB) as well as *Alchornea cordifolia* leaves (ACL) and bark (ACB) had respective IC_50_ values of 0.98 μg/mL, 1.45 μg/mL, 8.02 μg/mL and 12.57 μg/mL in CCRF-CEM cells. They were further tested in 8 other cell lines as well as in normal AML12 hepatocytes. IC_50_ values ranging from 2.71 μg/mL (towards glioblastoma U87MG.Δ*EGFR* cells) to 10.30 μg/mL (towards breast adenocarcinoma MDA-MB-231-*BCRP* cells) for AAB, from 3.43 μg/mL (towards U87MG cells) to 10.77 μg/mL (towards colon carcinoma HCT116 (*p53*^*−/−*^) cells) for AAR and from 0.11 μg/mL (towards CCRF-CEM cells) to 108 μg/mL (towards leukemia CEM/ADR5000 cells) for doxorubicin (as control drug) were obtained. ACL and ACB extracts displayed selective activities. AAR and ACL extracts induced apoptosis in CCRF-CEM cells, through caspases activation and loss of MMP, while apoptotic cell death was mediated by MMP diruption and increase ROS production for ACL.

**Conclusion:**

Some of the tested plants namely *Albizia adianthifolia, Alchornea cordifolia, Alchornea laxiflora, Pennisetum purpureum, Spathodea campanulata* represent a potential source of novel anticancer drugs. Especially, *Albizia adianthifolia* and *Alchornea cordifolia* revealed considerable cytotoxic activities that could be exploited to develop phytomedicines to fight cancers including MDR phenotypes.

## Background

Malignant diseases are amongst the major causes of death worldwide with a growing burden and low survival rates in developing countries [1]. Clinically, chemotherapy is still hampered by treatment failures due to multidrug resistance (MDR) of cancer cells. Anticancer drug development should take into account the fact that cancer cells rapidly acquire resistance [2–5]. Natural ressources such as medicinal plants constitute an indeniable reservoir of antiproliferative compounds [6]. Hence, fighting cancers and mostly drug-resistant phenotypes with phytochemical represents a very promising alternative, especially regarding the diversity of plant’s secondary metabolites. In the past, several bioactive compounds belonging to several classes of secondary metabolites isolated from African plants showed considerable antiproliferative activity against MDR cancer cells. Some of these molecules include benzophenones (2,2′,5,6′-tetrahydroxybenzophenone, guttiferone E, isogarcinol and isoxanthochymo) [7], xanthones (xanthone V1, quinones: 2-acetylfuro-1,4-naphthoquinone) [8], flavonoids (gancaonin Q, 4-hydroxylonchocarpin, 6-prenylapigenin, 6,8-diprenyleriodictyol [9], 2′,4′-dihydroxy-3′,6′-dimethoxychalcone, 4′-hydroxy-2′,6′-dimethoxychalcone, cardamomin [10, 11], 8-hydroxycudraxanthone G, morusignin I and cudraxanthone I [12] and alkaloids (isotetrandrine [13], montrofoline, 1-hydroxy-4-methoxy-10-methylacridone, norevoxanthine, evoxanthine and 1,3-dimethoxy-10-methylacridone) [14]. Moreover, several African medicinal plants previously displayed good cytotoxicity towards drug-sensitive and drug-resistant cancer cell lines. These plants include *Echinops giganteus, Xylopia aethiopica, Piper capense, Imperata cylindrica* [15, 16], *Beilschmiedia acuta, Clausena anisata, Fagara tessmannii, Newbouldia laevis, Polyscias fulva* [17], *Garcina lucida, Fagara heitzii, Hymenocardia lyrata* [18], *Gladiolus quartinianus, Vepris soyauxii* and *Anonidium mannii* [19].

In our ongoing search of anticancer drugs from African medicinal plants, we undertook the present work to assess the cytotoxicity of 10 Cameroonian medicinal plants traditionally used to manage cancer or disease states bearing relevance to cancer or cancer-like symptoms, such as immune and skin disorders, inflammatory, infectious, parasitic and viral diseases [15]. The study was extended to the evaluation of the ability of extracts from two most active plants, *Albizia adianthifolia* and *Alchornea cordifolia* to alter the cell cycle distribution, caspases activity, mitochondrial membrane potential (MMP) and to increase reactive oxygen species (ROS) in leukemia CCRF-CEM cells.

## Methods

### Plant material and extraction

All medicinal plants parts used in the present study were collected in different regions of Cameroon in January 2014. These included leaves, bark and roots of *Alchornea cordifolia*, *Alchornea laxiflora*, *Albizia adianthifolia* and *Spathodea campanulata*, leaves and roots of *Combretum hispidum* and *Laportea ovalifolia* and the whole plant of *Boerhavia diffusa*, *Eremomastax speciosa*, *Laportea aestuans* and *Pennisetum purpureum*. The plants were identified at the National Herbarium (Yaoundé, Cameroon), where voucher specimens were deposited under the reference numbers shown in Table 1. The air-dried and powdered plant material was soaked in methanol for 48 h, at room temperature. The methanol extract was concentrated in vacuum under reduced pressure to give the crude extract. This extract was then conserved at 4 °C until further use.

**Table 1:** Published bioactivity and phytochemistry of the studied plants

Species (family); Voucher Number^a^	Traditional uses	Parts used (% yield)^b^	Bioactive or potentially bioactive components	Bioactivity of crude extract
*Albizia adianthifolia (Schum.)* (Fabaceae); 24729/SRF/Cam	Treatment skin diseases, bronchitis, inflamed eyes, tapeworm, headaches and sinusitis [33, 34]	Leaves (9.88), bark (10.65) and roots (7.12)	Adianthifoliosides A, B, D [30, 35], lupeol and aurantiamide acetate [32], prosapogenins [31]	Antimicrobial effect of crude extract against *Ec, Ef, Pa, Pm, Kp, Sa, Sf, St, Ca, Ct, Ck, Cg, Cl, Cn* [32, 36]; aqueous extract: antioxidant [32, 37]
*Alchornea cordifolia* (Schum. & Thonn.) Müll.-Arg. (Euphorbiaceae); 9657/SRF/Cam	Treat rheumatic pains, fever, wounds, diarrhea, convulsions, coughs, gonorrhoea, yaws, ulcer, rheumatic pains, bronchial troubles [38, 39]	Leaves (7.84), bark (11.32) and roots (6.23)	Alchornine, alchorneinone, gentisnic acid and yohimbine [38]	Crude extract showed: spasmolytic [38], anti-inflammatory [40], anti-microbial [41],[42], anti-diarrheal [43] and analgesic [44] activities
*Alchornea laxiflora* (Benth.) Pax & K Hoffm. (Euphorbiaceae); 9661/SRF/Cam	Treatment of inflammatory and infectious diseases, poliomyelitis and measles. [45, 46]	Leaves (10.21), bark (13.17) and roots (5.58)	Quercetin-7,4′-disulphate, quercetin, quercetin-3′,4′-disulphate, quercetin-3,4′-diacetate, rutin and quercetrin [45]	Methanol fraction of leave extracts : antimicrobial on *Ba, Bc, Ec, Kp, Pa, Pf, Sa, Ag, Af, As, Ca, Cp* [47]^,^[36]; Crude extract : antioxidant [48]
*Boerhavia diffusa* Lin (Nyctaginaceae); 15247/SRF/Cam	Treatment of diabetes, asthma, bronchial infections [49]	Whole plant (11.42)	Boeravinones G, H [50]	Crude extract of leaves: Antioxidant and hepatoprotective properties [51], antimicrobial activity of crude extract agains*: Pa, Ec, St, Sf* [52], *Kp, Ea, Ps* [36]
*Combretum hispidum* Laws (Combretaceae); 48289/HNC	Treatment of stomach aches, diarrhea, gastro-intestinal disorders, liver complaints, skin infections, urinary tract infections [53–55]	Leaves (14.32), roots (8.47)	Alkaloids, polyphenols, flavonoids, anthraquinones, coumarins, tannins, triterpenes, sterols ans saponins [36]	Crude extract of bark: anti-hepatoxic, anti-inflammatory, antiparasitic, mollucidal effect [56], Antimicrobial effect of crude extract against *Ec*, *Kp, Pa, Ea*, *Ps* [36]
*Eremomastax speciosa* (Hochst.) Cufod. (Acanthaceae); 24165/SRF/Cam	Haematopoietic, antidiarrhoeal, antiulcer, treatment of female infertility, dysentery, anemia, irregular menstruation, fracture, hemorrhoids and urinary tract infections [57, 58]	Whole plant (14.69)	Alkaloids, polyphenols, flavonoids, tannins, triterpenes, sterols ans saponins [42]	Antimicrobial effect of crude extract against *Ec, Sa, Ca* [59], *Ec*, *Pa, Ea*, *Ps* [42]
*Laportea aestuans* (Linn.) Chew (Urticaceae); 34812/HNC	Antihelmintic, treatment of headache, syphilitic yaws, fever, gonorrhea, rheumatism, menopausal disorder, antidote, asthma, hypertension, stomachage, diarrhea, wounds [60]	Whole plant (8.82)	Vanilic acid, gallic acid, ferulic acid, (6)-gingerol, capsaicin, rosemamic acid, tannic acid, p-coumaric acid, caffeic acid, scopoletin, catechin, resveratrol, genistein, apigenin, kaempherol, epicatechin, epigallocathechin, ellagic acid, myricitin acid, quercetin- 3, 7, 4-trimethyl ether, quercetin–3,7,3′,4′-trimethyl ether, artemetin, kaempferol–arabinoside, quercitrin, isoquercitrin, naringin, rutin, hesperidin, limonene, α-pinene, β-pinene, cis-ocimene, myrcene, citronellol, neryl acetate, malvidine, lycopene, carotene, lutein, hispogenin, diosgenin, neochlorogenin, hecogenin, [61, 62]	Antimicrobial effect of crude extract against; *K, Ec, Sa, Bs, St, Pa, Ca,* [61, 63] *Ec*, *Pa, Kp, Ea*, *Ps* [42]
*Laportea ovalifolia* (Schum.) Chew (Urticaceae) 44306/HNC	Treatment of headache, internal ulcers, diabetes, bronchitis and wired [64, 65]	Leaves (12.13) and roots (6.17)	Laportoside A and laportomide A [66]	Crude extract of leaves: antidiabetic and hypolipidaemic effects [65], and crude extract against *Ec*, *Kp, Pa, Ea*, *Ps* [36]
*Pennisetum purpureum* Schumach. (Poaceae); 12525/SRF/Cam	Diuretic, antivenomous, treatment of measles, wound healing [67]	Whole plant (8.82)	Alkaloids, polyphenols, flavonoids, tannins, triterpenes, sterols ans saponins [42]	Antimicrobial effect of EO: *Ec, Pa* [67] and crude extract against *Ec*, *Kp, Pa, Ea*, *Ps* [42]
*Spathodea campanulata P.* Beauv. (Bignoniaceae) 22791/SRF/Cam	Treatment of mental disorders, malaria, hemorrhoids, bacterial infections, HIV, poor blood circulation, gastro-intestinal diseases, urinary tract disorders [68]	Leaves (16.13), bark (15.27) and roots (11.41)	Carbohydrates, alkaloids, tannins, iridoid glucoside, phydroxy-benzoic acid, methyl p-hydroxy-benzoate [68, 69]	Antimicrobial effect of against *Pv, Ec, Kp* [70], *Pa, Ea*, *Ps* [42]

^a^(HNC): Cameroon National Herbarium; (SRF/Cam):*Société des Réserves Forestières du Cameroun*; ^b^yield calculated as the ratio of the mass of the obtained methanol extract/mass of the plant powder; *EO* Essential oil, *As Aspergillus niger, Ag Aspergillus glaucus, Af Aspergillus flavus, Ba Bacillus anthracis, Bc Bacillus cereus, Bs Bacillus subtilis, Ca Candida albicans, Cg Candida glabrata, CK Candida krusei, Cl Candida lusitaniae, Cn Cryptococcus neoformans, Cp Candida pseudotropicalis, Ct Candida tropicalis, Ec Escherichia coli, Ea Enterobacter aerogenes, Ef Enterococcus faecalis, Kp Klebsiella pneumoniae, Pa Pseudomonas aeruginosa, Pf Pseudomonas fluorenscens, Pm Proteus mirabilis, Pv Proteus vulgaris, Ps Providencia stuartii, Sa Staphylococcus aureus, Sf Shigella flexneri, Se Salmonella enterica, St Salmonella typhi;* Underline: disease states bearing relevance to cancer or cancer-like symptoms

### Chemicals

Doxorubicin 98.0 % and vinblastine ≥ 96 % from Sigma-Aldrich (Munich, Germany) were provided by the University Pharmacy of the Johannes Gutenberg University (Mainz, Germany) and dissolved in phosphate buffer saline (PBS; Invitrogen, Eggenstein, Germany) at a concentration of 10 mM. Geneticin > 98 % (Sigma-Aldrich) was stored at a stock concentration of 72.18 mM.

### Cell cultures

The cell lines used in the present study, their origins and their treatments were previously reported. They include drug-sensitive leukemia CCRF-CEM and multidrug-resistant P-glycoprotein-over-expressing subline CEM/ADR5000 cells [4, 20, 21], breast cancer MDA-MB-231-pcDNA3 cells and its resistant subline MDA-MB-231-*BCRP* clone 23 [22], colon cancer HCT116 (*p53*^*+/+*^) cells and its knockout clone HCT116 (*p53*^*−/−*^), glioblastoma U87MG cells and its resistant subline U87MG.Δ*EGFR* [7, 12, 16]. Leukemia CCRF-CEM and CEM/ADR5000 cells were cultured in RPMI 1640 medium (Invitrogen) supplemented with 10 % fetal calf serum in a humidified 5 % CO_2_ atmosphere at 37 °C. This medium was also used for the cytotoxicity test with these two cell lines. MDA-MB-231-*BCRP* as well as U87MG.Δ*EGFR* and HCT116 *(p53−/−)* were maintained in DMEM medium containing 10 % FBS (Invitrogen) and 1 % penicillin (100 U/mL)-streptomycin (100 μg/mL) (Invitrogen) and were continuously treated with 800 ng/mL and 400 μg/mL geneticin, respectively. The cytotoxicity of all carcinoma cells was performed in DMEM medium containing 10 % FBS (Invitrogen) and 1 % penicillin-streptomycin.

### Resazurin reduction assay

The cytotoxicity of the tested samples was performed by resazurin reduction assay as previously described [7, 23]. The assay is based on reduction of the indicator dye, resazurin, to the highly fluorescent resorufin by viable cells. Non-viable cells rapidly lose the metabolic capacity to reduce resazurin and thus produce no fluorescent signal. Briefly, adherent cells were detached by treatment with 0.25 % trypsin/EDTA (Invitrogen) and an aliquot of 1x10^4^ cells was placed in each well of a 96-well cell culture plate (Thermo Scientific, Germany) in a total volume of 200 μL. Cells were allowed to attach overnight and then were treated with different concentrations of the studied sample. For suspension cells, aliquots of 10^4^ cells per well were seeded in 96-well-plates in a total volume of 100 μL. The studied sample was immediately added in varying concentrations in an additional 100 μL of culture medium to obtain a total volume of 200 μL/well. After 24 h or 48 h, 20 μL resazurin (Sigma-Aldrich, Germany) 0.01 % w/v in ddH_2_O was added to each well and the plates were incubated at 37 °C for 4 h. Fluorescence was measured on an Infinite M2000 Pro™ plate reader (Tecan, Germany) using an excitation wavelength of 544 nm and an emission wavelength of 590 nm. Each assay was done at least two times, with six replicates each. The viability was evaluated based on a comparison with untreated cells. IC_50_ values represent the sample’s concentrations required to inhibit 50 % of cell proliferation and were calculated from a calibration curve by linear regression using Microsoft Excel [8, 11]. The contribution of the extracts (at various tested concentrations) to the fluorescence has been determined, both in the absence and presence of resazurin, prior to any cell studies. In a preliminary study, all samples were tested against the sensitive CCRF-CEM cells at various concentrations ranging from 0.63 to 80 μg/mL (crude extracts) or 0.08 to 10 μg/mL (doxorubicin), and samples displaying IC_50_ values below 20 μg/mL were further investigated in 8 other tumor cell lines as well as in normal AML12 hepatocytes. Doxorubicin was used as positive control, while dimethylsulfoxide (DMSO) used to dissolve the samples was used as negative control. The highest concentration of DMSO was less than 0.4 %.

### Flow cytometry for cell cycle analysis and detection of apoptotic cells

Extracts from *Albizia adianthifolia* roots (AAR) and *Alchornea cordifolia* leaves (ACL) that displayed the best cytotoxicity as well as doxorubicin were used to treat CCRF-CEM cells (1 × 10^6^) at their IC_50_ values. The cell cycle was then analyzed after incubation for 24 h, 48 h and 72 h. All reagents, experimental conditions and apparatus were identical to those previously reported [12, 16]. Briefly, cell cycle analysis was performed by flow cytometry using Vybrant® DyeCycle™ (Invitrogen, Darmstadt, Germany). Cells were measured after Vybrant® DyeCycle™ Violet staining (30 min at 37 °C) on a LSR-Fortessa FACS analyzer (Becton-Dickinson, Heidelberg, Germany) using the violet laser. Vybrant® DyeCycle™ Violet stain was measured with 440 nm excitation. Cytographs were analyzed using FlowJo software (Celeza, Switzerland). All experiments were performed at least in triplicate.

### Caspase-Glo 3/7, caspase-Glo 8 and caspase-Glo 9 assay

Caspase activity in CCRF-CEM cells treated with AAR and ACL was detected using Caspase-Glo 3/7, Caspase-Glo 8 and Caspase-Glo 9 Assay kits (Promega, Mannheim, Germany) as previously described [24].

### Analysis of mitochondrial membrane potential (MMP)

CCRF-CEM cells were treated with AAR, ACL or vinblastine. The MMP was analyzed using 5,5′,6,6′-tetrachloro-1,1′,3,3′-tetraethylbenzimidazolylcarbocyanine iodide) (JC-1; Biomol, Hamburg, Germany) staining as previously described [19]. Cells were measured in a LSR-Fortessa FACS analyzer (Becton-Dickinson). The JC-1 signal was measured at an excitation of 561 nm (150 mW) and detected using a 586/15 nm band-pass filter. The signal was analyzed at 640 nm excitation (40 mW) and detected using a 730/45 nm bandpass filter. Cytographs were analyzed using FlowJo software (Celeza, Olten, Switzerland). All experiments were performed at least in triplicate.

### Measurement of reactive oxygen species (ROS) by flow cytometry

The 2′,7′-dichlorodihydrofluorescein diacetate (DCFH-DA) (Sigma-Aldrich, Schnelldorf, Germany) was used for the detection of ROS in CCRF-CEM cells treated with AAR, ACL, DMSO (solvent control), or hydrogen peroxide (H_2_O_2_; positive control) as previously described [17, 25, 26]. Cells were measured in a FACS Calibur flow cytometer (Becton-Dickinson). Dichlorofluorescein (DCF) was measured at 488 nm excitation (25 mW) and detected using a 530/30 nm band-pass filter. Cytographs were analyzed using FlowJo software (Celeza). All experiments were performed at least in triplicate.

### Statistical analysis

Statistical analysis of all data was performed using a Student’s *t*-test or Kruskal–Wallis test followed by Dunn’s post-hoc multiple comparison test (Graph-Pad Prism 5.01; GraphPad Software, Inc., CA, USA). *P* < 0.05 denoted significance in all cases.

## Results

In the present investigation, the cytotoxicity of 20 methanol extracts from 10 plants was first determined at different concentrations in drug-sensitive CCRF-CEM leukemia cells. The results are summarized in Table 2. Twelve out of 20 (60 %) extracts displayed IC_50_ values below 80 μg/mL. These extracts were from *Pennisetum purpureum*, *Spathodea campanulata* bark, *Spathodea campanulata* roots, *Alchornea laxiflora* bark, *Alchornea laxiflora* leaves, *Albizia adianthifolia* leaves (AAL), *Combretum hispidum* leaves, *Alchornea cordifolia* roots (ACR), *Alchornea cordifolia* bark (ACB), *Alchornea cordifolia* leaves (ACL), *Albizia adianthifolia* bark (AAB) and *Albizia adianthifolia* roots (AAR). Extracts from *Alchornea laxiflora* roots, *Boerhavia diffusa* (whole plant), *Combretum hispidum* bark, *Eremomastax speciosa* (whole plant), *Laportea aestuans* (whole plant), *Laportea ovalifolia* leaves, *Laportea ovalifolia* roots, *Spathodea campanulata* leaves resulted in more than 50 % proliferation of CCRF-CEM cells at 80 μg/mL (Table 2). Four extracts from two plants including ACB (IC_50_ value of 12.57 μg/mL), ACL (IC_50_: 8.02 μg/mL), AAB (IC_50_: 1.45 μg/mL) and AAR (IC_50_: 0.98 μg/mL) as well as doxorubicin (IC_50_: 0.11 μg/mL) displayed IC_50_ values below 20 μg/mL in CCRF-CEM cells (Table 2). These extracts were further selected for IC_50_ determination towards a panel of sensitive and MDR cell lines. The results summarized in Table 2 revealed IC_50_ values ranging from 2.71 μg/mL (towards glioblastoma U87MG.Δ*EGFR* cells) to 10.30 μg/mL (towards breast adenocarcinoma MDA-MB-231-*BCRP* cells) for AAB, from 3.43 μg/mL (towards U87MG cells) to 10.77 μg/mL (towards resistant colon carcinoma HCT116 (*p53*^*−/−*^) cells) for AAR and from 0.11 μg/mL (towards CCRF-CEM cells) to 108 μg/mL (towards P-glycoprotein-over-expressing CEM/ADR5000 cells) for doxorubicin on the 8 other cancer cell lines studied. Extracts from *Alchornea cordifolia,* ACL and ACB displayed selective activities. However, ACL and ACB were also less toxic towards normal AML12 hepatocytes, with IC_50_ values above 80 μg/mL contrary to AAB (IC_50_: 29.18 μg/mL) and AAR (IC_50_: 29.14 μg/mL). It is worth noting that collateral sensitivity (or hypersensitivity: higher toxicity to resistant than to sensitive cells with a degree of resistance below 1) was observed in drug-resistant epidermal growth factor receptor-transfected U87MG.Δ*EGFR* cells to AAB (degree of resistance of 0.43-fold), to AAR (0.39-fold), to ACL (0.83-fold) and to ACB (<0.40-fold) compared to its sensitive counterpart U87MG cells. Importantly, if cross-resistance to the tested extracts were observed, the degrees of resistance were in all cases lower than that of the reference compound, doxorubicin (Table 3). AAR and ACL were the most active extracts from *Albizia adiathifolia* and *Alchornea cordifolia* respectively, and were subsequently used for mechanistic studies.

**Table 2:** IC_50_ values (after 72 h) of the tested plant extracts towards leukemia CCRF-CEM cells and as determined by the resazurin assay

Tested plant	IC_50_ values (μg/mL)
*Alchornea laxiflora roots*	>80
*Boerhavia diffusa (whole plant)*	>80
*Combretum hispidum bark*	>80
*Eremomastax speciosa (whole plant)*	>80
*Laportea aestuans (whole plant)*	>80
*Laportea ovalifolia leaves*	>80
*Laportea ovalifolia roots*	>80
*Spathodea campanulata leaves*	>80
*Pennisetum purpureum (whole plant)*	69.01 ± 7.99
*Spathodea campanulata bark*	63.29 ± 8.61
*Spathodea campanulata roots*	58.08 ± 4.27
*Alchornea laxiflora bark*	49.21 ± 11.16
*Alchornea laxiflora leaves*	43.67 ± 4.06
*Albizia adianthifolia leaves* (AAL)	33.34 ± 3.55
*Combretum hispidum leaves*	32.05 ± 2.41
*Alchornea cordifolia roots* (ACR)	27.28 ± 1.22
*Alchornea cordifolia bark* (ACB)	**12.57 ± 0.55**
*Alchornea cordifolia leaves* (ACL)	**8.02 ± 0.23**
*Albizia adianthifolia bark* (AAB)	**1.45 ± 0.13**
*Albizia adianthifolia roots* (AAR)	**0.98 ± 0.08**
Doxorubicin	**0.11 ± 0.03**

In bold: significant cytotoxic effect; Leukemia CCRF-CEM cells were tested in RPMI 1640 medium supplemented with 10 % FBS and 1 % penicillin-streptomycin

**Table 3:** Cytotoxicity of plant extracts and doxorubicin towards sensitive and drug-resistant cancer cell lines and normal cells as determined by the resazurin assay after 72 h

Cell lines	Samples, IC_50_ values in μg/mL and degrees of resistance^a^ (in brackets)				
	Extracts				Doxorubicin
	AAB	AAR	ACL	ACB	
CEM/ADR5000	**6.53 ± 0.77** (4.50)	**6.52 ± 0.57** (6.65)	22.57 ± 2.17 (2.81)	34.87 ± 3.91 (2.77)	108.00 ± 7.92 (975.60)
MDA-MB-231-*pcDNA*	**5.99 ± 0.3**	**3.79 ± 0.44**	29.51 ± 3.08	25.54 ± 3.01	**0.61 ± 0.15**
MDA-MB-231-*BCRP* Degree of resistance	**10.30 ± 1.22** (1.72)	**8.00 ± 1.01** (2.11)	35.42 ± 3.35 (1.20)	33.60 ± 2.14 (1.32)	**4.33 ± 0.26** (7.12)
HCT116 (*p53* ^*+/+*^)	**5.13 ± 0.64**	**4.40 ± 0.32**	33.15 ± 2.87	29.07 ± 2.68	**0.78 ± 0.16**
HCT116 (*p53* ^*−/−*^) Degree of resistance	**9.46 ± 0.88** (1.84)	**10.77 ± 0.79** (2.45)	>80 (>2.41)	>80 (2.75)	**2.25 ± 0.04** (2.88)
U87MG	**6.29 ± 0.40**	**8.86 ± 0.69**	38.63 ± 3.32	>80	**0.59 ± 0.08**
U87MG.Δ*EGFR* Degree of resistance	**2.71 ± 0.35** (0.43)	**3.43 ± 0.48** (0.39)	32.12 ± 1.19 (0.83)	31.84 ± 2.36 (<0.40)	**3.38 ± 0.32** (5.76)
HepG2 Degree of resistance	**7.54 ± 0.55** (3.87)	**6.84 ± 0.64** (4.26)	>80	>80	**2.12 ± 0.52** (>37.74)
AML12	29.18 ± 3.03	29.14 ± 2.17	>80	>80	>80

^a^The degree of resistance was determined as the ratio of IC_50_ value in the resistant divided by the IC_50_ in the sensitive cell line; CEM/ADR5000, MDA-MB-231-*BCRP,* HCT116 (*p53* ^*−/−*^), U87MG.Δ*EGFR* and AML12 were used as the corresponding resistant counterpart for CCRF-CEM (Table 1), MDA-MB-231-*pcDNA*, HCT116 (*p53* ^*+/+*^), U87MG and HepG2 cells, respectively. The tested methanol extracts were from *AAB Albizia adianthifolia bark, AAR Albizia adianthifolia roots, ACL Alchornea cordifolia leaves, ACB Alchornea cordifolia bark.* In bold: significant cytotoxic effects. Leukemia CEM/ADR5000 cells were tested in RPMI 1640 medium while carcinoma cells tested using DMEM medium, both containing 10 % FBS and 1 % penicillin-streptomycin

**Fig. 1:**
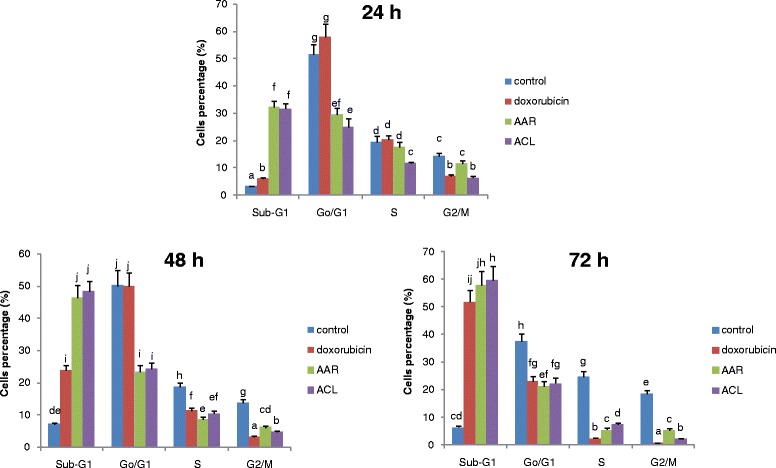
Cell cycle distribution of CCRF-CEM leukemia cells treated with extracts from *Albizia adianthifolia* roots (AAR), *Alchornea cordifolia* leaves (ACL) or doxorubicin (control drug) at different time. AAR and ACL were tested at 0.98 μg/mL and 8.02 μg/mL respectively, while doxorubicin was tested at 0.11 μg/mL corresponding to their IC_50_ values. At each experimental time (24 h, 48 h, 72 h), data with different superscript letters are significantly different (*P* < 0.05)

**Fig. 2:**
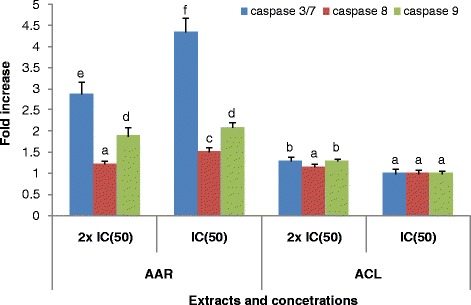
Enzymatic activity of caspase 3/7, caspase 8 and caspase 9 after 6 h treatment of CCRF-CEM cells with AAR and ACL extracts. The activity of caspase is expressed as percentage % relative to untreated cells. IC_50_: 0.98 μg/mL and 8.02 μg/mL for AAR and ACL extracts, respectively. Data with different superscript letters are significantly different (*P* < 0.05)

**Fig. 3:**
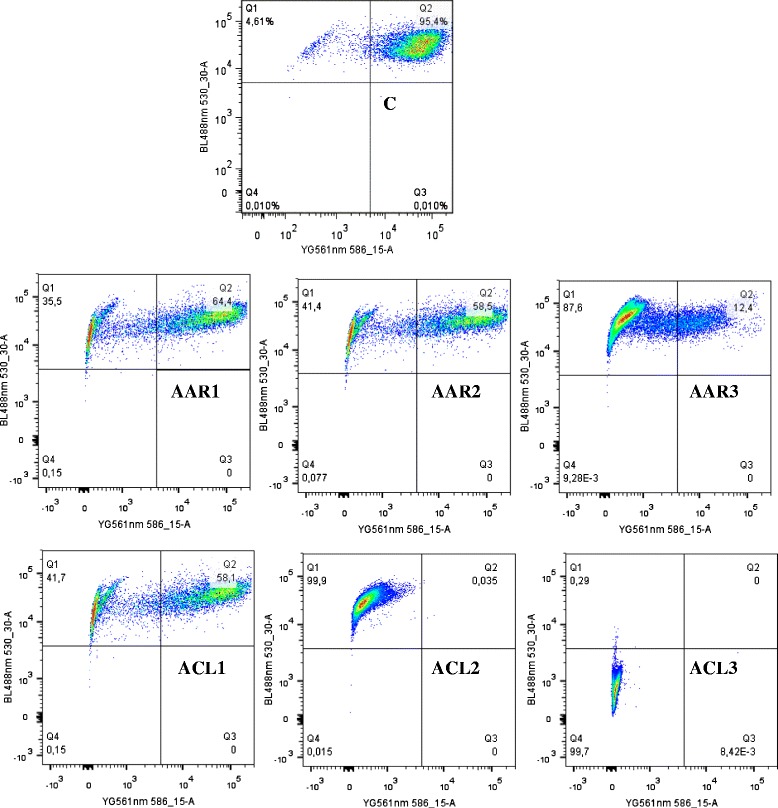
Effect of AAR and ACL extracts on the mitochondrial membrane potential in CCRF-CEM cells. C: control; AAR was tested at 24 h at 0.49 μg/mL (AAR1), 0.98 μg/mL (AAR2), and 1.96 μg/mL (AAR3), while ACL was tested at 4.01 μg/mL (ACL1), 8.02 μg/mL (ACL2), and 16.04 μg/mL (ACL3) corresponding to 1/2-fold, IC_50_ and 2-fold IC_50_ values. Data for the positive control, vinblastine under similar experimental conditions were previously reported [7]. Loss of MMP (Q1), intact cells (Q2), ruptured cell membrane (Q3 and Q4)

**Fig. 4:**
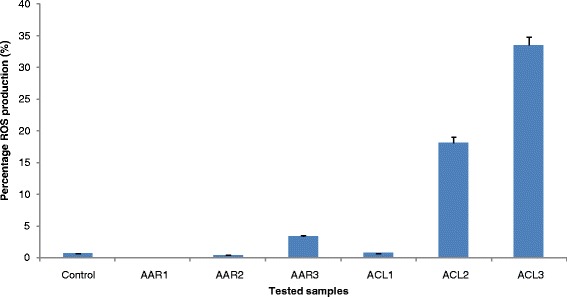
ROS generation in CCRF-CEM cells after treatment with AAR and ACL extracts for 24 h. C: control; AAR was tested at 24 h at 0.49 μg/mL (AAR1), 0.98 μg/mL (AAR2), and 1.96 μg/mL (AAR3), while ACL was tested at 4.01 μg/mL (ACL1), 8.02 μg/mL (ACL2), and 16.04 μg/mL (ACL3) corresponding to 1/2-fold, IC_50_ and 2-fold IC_50_ values

IC_50_ values of AAR and ACL extracts as well as doxorubicin were used to treat CCRF-CEM cells for 6 h, and the cycle distribution was analyzed. The results are depicted in Fig. 1. Dose-dependent and significant modifications of the cell cycle phases were observed. Both AAR and ACL induced cell cycle arrest in the G0/G1 phase. After treatment with these two extracts, CCRF-CEM cells underwent apoptosis with a dose-dependent increase in the sub-G0/G1 phase. The percentages of cells in the sub-G0/G1 phase varied from 32.14 % (in 24 h) to 57.99 % (72 h) and from 31.69 % (24 h) to 59.67 % (72 h), respectively, for AAR and ACL treatments, while doxorubicin increased apoptosis in a range of 6.02 % (24 h) to 51.87 % (72 h). The highest percentage of sub-G0/G1 phase in non-treated cells was only 6.42 % after 72 h. After treating CCRF-CEM cells for 6 h at 2-fold IC_50_, AAR induced 4.35-fold, 2.02-fold and 1.52-fold increase of caspase 3/7, caspase 9 and caspase 8 activities, respectively, whereas no changes were observed upon ACL treatment (Fig. 2). AAR also induced significant MMP loss in a range of 35.5 % (1/2-fold IC_50_ treatment) to 87.6 % (2-fold IC_50_) (Fig. 3). ACL caused up 41.7 % MMP loss at 1/2-fold IC_50_ treatment and complete rupture of the membrane (99.7 %) at 2-fold IC_50_ (Fig. 3). A 48.6 % loss of MMP at 2-fold IC_50_ of vinblastine was previously reported under similar experimental conditions in CCRF-CEM cells [12]. AAR did not induce ROS generation in CCRF-CEM cells contrary to ACL (Fig. 4). Dose-dependant increase in ROS production was also observed upon treatment of cells with ACL in a range of 0.73 % (1/2-fold IC_50_ treatment) to 33.6 % (2-fold IC_50_).

## Discussion

The development of resistance by malignant cells remains a serious issue in cancer chemotherapy. Cancer cells rapidly develop chemoresistance, mainly due to the presence of adenosine triphosphate-binding cassette (ABC) transporters [2–4], such as the breast cancer resistance protein (BCRP/ABCG2) and P-glycoprotein (P-gp/MDR1/ABCB1) [2] as well as the oncogene epidermal growth factor receptor (EGFR) [3, 4, 27] and the deletion or inactivation of tumor suppressor gene p53 [5]. Hence, identifying the mechanisms of resistance to different drugs is necessary, in order to efficiently prevent and overcome drug resistance. In this study, multi-factorial drug-resistant cancer cell lines such as leukemia CEM/ADR5000 cells over-expressing P-glycoprotein, breast adenocarcinoma MDA-MB-231-*BCRP* clone 23 expressing BCRP, p53 knockout HCT116 (*p53*^*−/−*^) colon cancer cells and EGFR-transfected U87MG.Δ*EGFR* glioblastoma cells [4, 7, 12, 20–22, 28] were used to determine to assess the cytotoxicity the selected plant extracts. According to the US NCI plant screening program, botanicals with IC_50_ values below of 20 μg/mL following incubation between 48 and 72 h [29] have been recognized as potential cytotoxic substances. In preliminary assays using the sensitive leukemia CCRF-CEM cells, AAB, AAR, ACL and ACB (Table 2) displayed IC_50_ values below 20 μg/mL and were therefore selected for further assays against MDR phenotypes of other cell lines. Interestingly, AAB and AAR also displayed IC_50_ values below or around 10 μg/mL and could therefore be considered as potential source for novel anti-cancer drugs. Most importantly, the degree of resistance of cells lines to AAB and AAR were in all cases lower than that of doxorubicin, highlighting their potential to combat MDR phenotypes. Though the IC_50_ values recorded with ACL and ACB were all above 20 μg/mL, the cytotoxicity of these two samples on malignant cells can still be considered interesting, as they were much less toxic on normal AML12 hepatocytes, highlighting their good selectivity. It is also worth to note that the two best extracts, AAB and AAR were slightly toxic to normal AML12 hepatocytes (IC_50_ values of 29.18 μg/mL and 29.14 μg/mL respectively for AAB and AAR). However, their high cytotoxicity towards cancer cells also suggests that they might be safely used in cancer chemotherapy. However, further evidence of the clinical efficacy of these extracts will be needed, as many phytochemicals are poorly bioavailable and they may be metabolized to more or less potent compounds by gut bacterial metabolism. MMP loss and increased ROS have been reported as a mode of apoptosis induction of plant extracts [29]. Hence, the ability of AAR and ACL to cause MMP breakdown in CCRF-CEM cells fits to this theory. The mode of action of AAR also includes the activation of caspases. Initiator caspases 9 (2.02-fold) and effector caspases 3/7 (4.35-fold) (Fig. 2) were significantly activated [29]. In addition to MMP alterations, ACL-induced apoptosis also include ROS production (Fig. 4).

To the best of our knowledge, the cytotoxicty of *Albizia adiathifolia* and *Alchornea cordifolia* towards the cell line panel tested in this study is being reported for the first time. Triterpenoid saponins such as adianthifoliosides A, B, and D isolated from *Albizia adianthifolia* exhibited cytotoxic effects towards Jurkat leukemia cells [30]. The presence of these compounds as well as other cytotoxic constituents such as prosapogenins [31] and aurantiamide acetate [32] found in *Albizia adianthifolia* could explain the antiproliferative effects of this plant.

## Conclusion

Twelve extracts from 5 medicinal plants (*Albizia adianthifolia, Alchornea cordifolia, Alchornea laxiflora, Pennisetum purpureum,* and *Spathodea campanulata*) displayed cytotoxicity against CCRF-CEM leukemia cells. They may represent a source for the development of novel anticancer drugs. Furthermore, *Albizia adianthifolia* and *Alchornea cordifolia* further displayed considerable cytotoxicity against MDR phenotypes in a panel of 8 other cancer cell lines. They may therefore be exploited to develop phytomedicine to fight cancers with various MDR phenotypes. AAR and AAB were the most cytotoxic extracts and the mechanism of AAR-induction apoptosis in CCRF-CEM cells included caspases activation and MMP loss. The mode of apotosis induction by ACL extract included MMP disruption and increased ROS generation in CCRF-CEM cells. The cytotoxicty of the two best plants, *Albizia adiathifolia* and *Alchornea cordifolia* towards the cell line panel tested in this study is being reported for the first time. Their purification will further be performed to identify their active constituents.

## Abbreviations

AAB, *Albizia adianthifolia* bark; AAL, *Albizia adianthifolia* leaves; AAR, *Albizia adianthifolia* roots; ABC, adenosine triphosphate-binding cassette; ACB, *Alchornea cordifolia* bark; ACL, *Alchornea cordifolia* leaves; ACR, *Alchornea cordifolia* roots; BCRP, breast cancer resistance protein; DCF, dichlorofluorescein; DCFH-DA, 2′,7′-Dichlorodihydrofluorescein diacetate; DMSO, dimethylsufoxide; EGFR, epidermal growth factor receptor; IC_50_, inhibitory concentration 50 %; JC-1, 5,5′,6,6′-tetrachloro-1,1′,3,3′-tetraethylbenzimidazolylcarbocyanine iodide; MDR, multi-drug resistant; MMP, mitochondrial membrane potential; PBS, phosphate buffer saline; ROS, reactive oxygen species
